# Tumor-Specific Antibody, Cetuximab, Enhances the *In Situ* Vaccine Effect of Radiation in Immunologically Cold Head and Neck Squamous Cell Carcinoma

**DOI:** 10.3389/fimmu.2020.591139

**Published:** 2020-11-12

**Authors:** Won Jong Jin, Amy K. Erbe, Ciara N. Schwarz, Abigail A. Jaquish, Bryce R. Anderson, Raghava N. Sriramaneni, Justin C. Jagodinsky, Amber M. Bates, Paul A. Clark, Trang Le, Keng-Hsueh Lan, Yi Chen, KyungMann Kim, Zachary S. Morris

**Affiliations:** ^1^ Department of Human Oncology, University of Wisconsin, Madison, WI, United States; ^2^ Department of Biostatistics and Medical Informatics, University of Wisconsin, Madison, WI, United States

**Keywords:** head and neck squamous cell carcinoma, EGFR, resistance, *in situ* vaccination, immunotherapy, immune checkpoint, cetuximab, radiation

## Abstract

In head and neck squamous cell carcinoma (HNSCC) tumors that over-expresses huEGFR, the anti-EGFR antibody, cetuximab, antagonizes tumor cell viability and sensitizes to radiation therapy. However, the immunologic interactions between cetuximab and radiation therapy are not well understood. We transduced two syngeneic murine HNSCC tumor cell lines to express human EGFR (MOC1- and MOC2-huEGFR) in order to facilitate evaluation of the immunologic interactions between radiation and cetuximab. Cetuximab was capable of inducing antibody-dependent cellular cytotoxicity (ADCC) in MOC1- and MOC2-huEGFR cells but showed no effect on the viability or radiosensitivity of these tumor cells, which also express muEGFR that is not targeted by cetuximab. Radiation enhanced the susceptibility of MOC1- and MOC2-huEGFR to ADCC, eliciting a type I interferon response and increasing expression of NKG2D ligands on these tumor cells. Co-culture of splenocytes with cetuximab and MOC2-huEGFR cells resulted in increased expression of IFNγ in not only NK cells but also in CD8+ T cells, and this was dependent upon splenocyte expression of FcγR. In MOC2-huEGFR tumors, combining radiation and cetuximab induced tumor growth delay that required NK cells, EGFR expression, and FcγR on host immune cells. Combination of radiation and cetuximab increased tumor infiltration with NK and CD8+ T cells but not regulatory T cells. Expression of PD-L1 was increased in MOC2-huEGFR tumors following treatment with radiation and cetuximab. Delivering anti–PD-L1 antibody with radiation and cetuximab improved survival and resulted in durable tumor regression in some mice. Notably, these cured mice showed evidence of an adaptive memory response that was not specifically directed against huEGFR. These findings suggest an opportunity to improve the treatment of HNSCC by combining radiation and cetuximab to engage an innate anti-tumor immune response that may prime an effective adaptive immune response when combined with immune checkpoint blockade. It is possible that this approach could be extended to any immunologically cold tumor that does not respond to immune checkpoint blockade alone and for which a tumor-specific antibody exists or could be developed.

## Introduction

Head and neck squamous cell carcinoma (HNSCC) carries a poor prognosis in patients with metastatic or recurrent disease (1, 2). Up to 90% of HNSCC tumors express the epidermal growth factor receptor (EGFR) (3, 4) and EGFR signaling plays a pivotal role in HNSCC cell proliferation (5, 6). Cetuximab is an antibody that binds to the extracellular domain of EGFR where it inhibits EGFR signaling and cell cycle progression and promotes apoptosis in HNSCC tumor cells (7, 8). Clinical studies demonstrate that cetuximab improves survival in patients with metastatic or recurrent HNSCC when combined with chemotherapeutics (9). Cetuximab also intrinsically sensitizes HNSCC cells to radiation therapy (10), and improves survival in patients with locally advanced HNSCC when used in combination with radiation (11, 12). Yet, most HNSCC patients respond only temporarily to cetuximab (9, 13, 14). This results from acquired resistance, despite persistent cetuximab binding to EGFR that is expressed on the tumor cell surface (15, 16). While acquired resistance limits the clinical benefit of cetuximab currently, an improved understanding of the impact of cetuximab on immune recognition of EGFR-expressing tumor cells may lead to development of novel therapeutic combinations for treating HNSCC patients.

Recent clinical data demonstrate that immune checkpoint inhibition with anti–PD-1 improves survival among patients with recurrent or metastatic HNSCC (17). With this treatment, a small percentage of patients with metastatic HNSCC may experience complete and durable tumor response. These results raise the possibility of dramatically improving survival and more consistently achieving curative outcomes for HNSCC patients by developing approaches to increase the rate and depth of response to anti–PD-1 immunotherapy. Immune checkpoint inhibitors are not typically effective in patients with immunologically “cold” tumors, characterized by low levels of T cell infiltrate and/or few mutation-created neo-antigens (18). In order to improve the response to immune checkpoint blockade in such cold tumors, others and we have been developing *in situ* cancer vaccine approaches (19). *In situ* vaccination is a therapeutic strategy that seeks to convert a patient’s own tumor into a nidus for enhanced presentation of tumor-specific antigens in a way that will stimulate and diversify an anti-tumor T cell response. The goal is localized destruction of a tumor to enable the destroyed cancer cells to function as a potent immune stimulus and personalized source of antigenicity for tumor-specific adaptive T cell immunity that is able to eradicate metastatic tumors.

Local radiation therapy can serve as a method of *in situ* vaccination. Recently, numerous case reports and retrospective studies have suggested safety and the potential for enhanced systemic anti-tumor response with combinations of radiation therapy and immune checkpoint blockade (20–27). Several prospective trials have also investigated the combination of radiation therapy and immune checkpoint blockade (28–32). These studies have further supported the safety of combining radiation and immune checkpoint inhibition and have demonstrated that radiation therapy can elicit an *in situ* vaccine effect when combined with immune checkpoint blockade clinically. For most tumor types, however, it remains to be determined whether and how radiation therapy can be used to elicit a clinically meaningful improvement in the duration, depth, or rate of response to immune checkpoint blockade. In the setting of head and neck cancer, a recently reported study randomized patients with metastatic HNSCC to receive either anti–PD-1 checkpoint blockade alone or in combination with radiation therapy to a single lesion (9 Gy × 3 fractions). The primary endpoint of objective response rate in non-irradiated lesions was not improved with combination therapy in that study (32).

Here, we evaluate a combined modality treatment approach to improve the *in situ* vaccine effect of radiation in HNSCC. To achieve this we combine: 1) radiation to enhance tumor cell immunogenicity, 2) the tumor-specific mAb, cetuximab, to enhance tumor destruction and antigen presentation by immune cells that express Fc-γ receptor (FcγR) including NK cells and macrophages, and 3) anti–PD-L1 immune checkpoint inhibition to augment and propagate an adaptive anti-tumor immune response.

## Material and Methods

### Cell Lines and Preparation

Wild-type (WT) MOC1 and MOC2 cells were a kind gift from Dr. Ravindra Uppaluri. huEGFR-expressing cells were generated by transduction of human EGFR (NM_005228.3) along with the puromycin resistance gene *via* lentivirus using pLV vectors designed in VectorBuilder. Stably transduced MOC1/2-huEGFR cells were selected with puromycin (4 μg/ml, Sigma-Aldrich) and single-cell cloned. MOC1/2-huEGFR cells were cultured in Dulbecco’s Modified Eagle Medium (DMEM; Corning)/Ham’s F12 (Corning) at a 2:1 mixture with 5% fetal bovine serum (Life Technologies), 1% penicillin/streptomycin (Life Technologies), 5 ng/ml epidermal growth factor (EGF; Gibco), 400 ng/ml hydrocortisone (Sigma-Aldrich), and 5 μg/ml insulin (Sigma-Aldrich). The human HNSCC cell line, SCC6, was cultured in DMEM containing 10% fetal bovine serum, 1 μg/ml hydrocortisone, and 1% penicillin/streptomycin. All cells were cultured in a humidified incubator at 37°C in an atmosphere of 5% carbon dioxide. ATCC guidelines were followed for authentication of all cell lines by monitoring morphology, growth curve analysis, and testing for mycoplasma (33).

### Cytotoxicity Assay

*In vitro* ^51^chromium (^51^Cr)-release cytotoxicity assay was performed as previously described (34). Briefly, “target” MOC1/2 and MOC1/2-huEGFR cells were labeled with ^51^Cr for 2 h and then washed and cultured with or without peripheral blood mononuclear cell (PBMC) “effectors” at indicated ratios (50:1, 40:1, 12.5:1, 10:1) in the presence or absence of cetuximab (0.5 μg/ml). After a 4 h incubation, the media was collected and the presence of ^51^Cr from lysed target cells was quantified using a beta counter (Packard Matrix 9600). The percent of lysis among target cells was calculated as 100 × (cetuximab treated − spontaneous)/(detergent lysed maximum − spontaneous).

### Clonogenic Assay of Radiation Sensitivity

We used a standard clonogenic assay to evaluate for effects of cetuximab on the radiosensitivity of MOC2-huEGFR cells. For this, we followed techniques that others have used to demonstrate the effect of cetuximab in sensitizing in HNSCC cells to radiation (10). Briefly, tumor cells were cultured for 24 h to allow the cells to adhere and then irradiated with indicated doses (0, 2, 4, and 8 Gy). The cells were then replated in the presence of non-specific human IgG or cetuximab (0.5 μg/ml) and allowed to grow for 5-7 days until the 0 Gy control group began forming colonies. The cells were then washed with PBS and stained using 0.5% crystal violet in methanol. Colonies consisting of 50 or more cells were counted, and the surviving fraction was determined as the (number of colonies)/(number of plated cells × plating efficiency).

### Murine Tumor Models

Mice were housed in accordance with the Guide for Care and Use of Laboratory Mice and treatments were performed under a protocol approved by the University of Wisconsin Institutional Animal Care and Use Committee. Mice aged 6–8 weeks were purchased from Taconic (C57BL/6, FcγR deficient C57BL/6.129P2-Fcer1g^tm1Rav^ N12).

MOC2 or MOC2-huEGFR tumor cells were engrafted by subcutaneous flank injection of 2x10^6^ tumor cells in 100 μl of PBS. Tumor sizes were measured using digital calipers and tumor volume was calculated as (width^2^ × length)/2. Treatment began when group tumor size reached 150 to 200 mm^3^, about 8 to 10 days after tumor cell implantation. The initial day of radiation treatment was defined as “day 1” for all experiments and for tumor response and survival curves. Intratumoral (IT) injections of non-specific human IgG (Sigma-Aldrich) or cetuximab (Eli Lilly) were administrated (50 μg/mouse) in 100 μl of PBS daily from day 6 to 10. Anti–PD-L1 antibody (B7-H1, BioXcell, 200 μg/mouse) was given *via* intraperitoneal (35) injection at days 0, 4, and 7. Animals were sacrificed when tumor volume exceeded a pre-determined maximum diameter (20 mm). To deplete NK cells, IP injections of 50 μg NK1.1 mAb (PK136, BioXcell) were given at days 0, 5, and 10.

### Radiotherapy

Radiation was delivered to tumor-bearing mice using a cabinet orthovoltage X-ray biological irradiator, X-RAD 320 (Precision X-Ray, Inc.). Local radiation to the tumor site was delivered after immobilization and shielding of mice using custom lead jigs that exposed only the tumor + ~5 mm margin. Radiation for *in vitro* experiments was delivered using an RS225 (Xstrahl) cabinet orthovoltage irradiator and was performed at least 24 h after plating the cells. Media was replaced immediately after radiation delivery.

### Immunohistochemistry and Cytokine Analysis

Mice engrafted and treated as above were sacrificed 48 h after treatment completion, and tumor specimens were collected. The tumors were embedded in OCT, flash frozen in liquid nitrogen, cryosectioned and placed on microscope slides. Tumor sections were fixed in cold acetone, rehydrated and blocked using 10% goat serum (Sigma-Aldrich) for 45 min. After washing, sections were incubated with mAb [CD8 (clone 53-6.7), NKG2A/C/E (clone 20d5), and FOXP3 (clone FJK-15s; all from eBioscience)] overnight in 1% goat serum. Following a wash, antigen-antibody complexes were labeled using an anti-rat IgG ImmPRESS kit (Vector Laboratories). The slides were developed with DAB substrate kit (Cell Signaling) for 60 s, counterstained with Mayer’s hematoxylin (Rowley Biochemical) for 30 s, then mounted with Permount (Fisher Chemical). All labeling was performed with primary control IgG antibody as a negative control. Digital pictures of the stained sections were taken at 200× magnification, and analyzed using ImageJ software. A minimum of three high-power field images were captured per tumor sample (n = 4–5 tumors/group). A blinded observer quantified positive labeled cells in each image.

Additional portions of tumor specimens were minced with a surgical blade and disaggregated using 5 mg of collagenase (Sigma-Aldrich) and 500 µg of DNase (Sigma-Aldrich) in 37°C incubator with shaking at 150 RPM for 30 min. Disaggregated tumor cells was strained through a 70 μm filter with 5 ml of RPMI. The samples were centrifuged at 2000 RPM for 5 min and the supernatants were collected. Using ELISA kits and following the manufacturer’s guidelines, cytokine concentrations IFNγ (BioLegend) in disaggregated tumor supernatants were measured using SpectraMax i3 at 450 nm absorbance.

### Cell Sorting and Flow Cytometry

Spleens from C57BL/6 mice were harvested, minced, and strained through a 70 μm filter in RPMI-1640 (Corning). Mice peripheral blood was collected from the submandibular vein. Red Blood Cell Lysing Buffer (Sigma-Aldrich) was added to the splenocytes to lyse erythrocytes. NK cells were sorted *via* negative selection using a magnetic-activated cell sorting (MACS) bead isolation kit (Miltenyi Biotec). To test intracellular IFNγ expression in splenocytes, MOC2-huEGFR cell (5 × 10^4^) were plated in 48-well plates for 24 h. The cells were radiated (8 Gy) and further cultured for 3 days. Splenocytes (1 × 10^6^) from WT or FcγR KO mouse were cocultured with radiated MOC2-huEGFR in the presence of cetuximab (2 μg/ml) for 24 h. The cells were treated GolgiStop™ protein transport inhibitor (BD Bioscience) for 5 h before antibodies staining. Total cells were harvested and treated CD16/32 antibody (BioLegend) for tumor cell non-specific binding.

Flow cytometry was performed using fluorescent beads (UltraComp Beads eBeads, Invitrogen, #01-2222-42) to determine compensation and fluorescence minus one (FMO) methodology to determine gating. Live cell staining was performed using Ghost Red Dye 780 (Tonbo Biosciences) according to the manufacturer’s instruction. Antibodies used for flow cytometry include: anti-CD45-PE-Cy7 (BioLegend), anti-CD3-FITC (BioLegend), anti-NK1.1-BV605 (BioLegend), anti-CD274 (PD-L1)-PE (BD Pharmingen), anti-IFNγ-APC (BioLegend) and Pan Rae1-APC (Miltenyi Biotec). In addition, human IgG (Sigma-Aldrich), cetuximab (Eli Lilly), calreticulin (ThermoFisher), and ULBP (ThermoFisher) were used as primary antibodies and anti-human IgG-PE (eBioscience), anti-rabbit IgG-PE (eBioscience), and anti-goat hamster IgG-PE (eBioscience) were used as a secondary antibody. After live-dead staining, a single cell suspension was labeled with the surface antibodies at 4°C for 30 min, washed three times using flow buffer (2% FBS + 2 mM EDTA in PBS). For intracellular staining, the cells were fixed and stained internal IFNγ with permeabilization solution according to the instruction (BD Cytofix/Cytoperm™). Flow cytometry was performed using an Attune NxT Flow Cytometer (ThermoFisher). Data was analyzed using FlowJo Software.

### Real-Time Quantitative PCR

After euthanizing mice, tumor specimens were collected and transferred to tubes containing ceramic beads (Fisher Brand) with 1 ml of Trizol reagent (ThermoFisher). Tumor tissue was homogenized using a Bead Ruptor Elite (OMNI) for 30 s. RNA was isolated using Qiagen’s RNeasy Mini Kit according to the manufacturer’s instructions. The RNA concentrations were determined using a Nanodrop 1000 spectrophotometer (ThermoScientific) and 2 μg of RNA was used to make cDNA using a QuantiTect Reverse Transcriptase Kit (Qiagen). Synthesized cDNA was diluted 1:10 with distilled water and qPCR was performed with 2 µl of diluted cDNA per reaction using the CFX96 Real-Time System (Bio-Rad) with PowerUp SYBR Green master mix (Applied Biosystems). Relative mRNA expression levels of target genes were determined according to the 2^−ΔΔCT^ method using HPRT as a reference gene (36). Primer sequences are listed in **Table 1**.

**Table 1:** List of primers.

Murine Genes	Primer sequences 5' -3'	
	Forward	Reverse
PD-L1	CCAGCCACTTCTGAGCATGA	CTTCTCTTCCCACTCACGGG
IFNβ	CCCTATGGAGATGACGGAGA	CTGTCTGCTGGTGGAGTTCA
IFNγ	AGCAAGGCGAAAAAGGATGC	TCATTGAATGCTTGGCGCTG
MHCI	TGTTCCCTGTGAGCCTATGG	GGAAGGGAAGACAGAGCAGT
MILL1	TCCCGAGATACAGGATTTCTGC	GCTGTGATCATTTTAGGCTGGC
MILL2	GTTGATCTTAGGGCTGCTCCTT	TGCTGGAACCATGAACCTCC
Rae1α	ATGGATACACCAACGGGCTG	TCCACTAAGCACTTCGCTTCA
Rae1δ	AAGAGGGGTGGCGATTTCAG	CTGGGCCCTCAGGGACTATT
H60b	GGTATTCGCTTGGTGTATGCTG	CTCCCCAGCACAGCTTGTTA
H60c	TCAACAAATCGTCGCCACAC	CCATCAAAGGGGCTGGACTT
ULBP1	TTGACAGTGCCTGAGACGTG	TCGTCTGAAGTCAACAGCACA
HPRT	AGCCTAAGATGAGCGCAAGT	GGCCACAGGACTAGAACACC
**Human Genes**	**Primer sequences 5' -3'**	
	**Forward**	**Reverse**
IFNβ	AAGGCCAAGGAGTACAGT	ATCTTCAGTTTCGGAGGTAA
HPRT	TATGGCGACCCGCAGCCCT	CATCTCGAGCAAGACGTTCAG

### Immunoblot and Cell Viability Assay

WT MOC1 and MOC2, MOC1- and MOC2-huEGFR, and SCC6 cells (5 × 10^5^ cells/well) were cultured in a 6-well plate in the absence or presence of human IgG or cetuximab (0.5 μg/ml) for 3 days and stimulated with EGF (30 ng/ml) for 5 min. To check γH2AX expression, cells were incubated with non-specific human IgG control or cetuximab (0.5 μg/ml) for 2 h and then irradiated (8 Gy). After 10 min, the cells were lysed and a Western blot was performed as previously described (37). Antibodies including anti-phospho-ERK (#9101), anti-ERK (#9102), anti-γH2AX (#9718), anti-GAPDH (#2118), and HRP-linked secondary antibodies were obtained from Cell Signaling Technologies.

To evaluate cell viability *in vitro*, cells (1 × 10^3^ cells/well) were cultured in a 96-well plate in the presence of varied concentrations of cetuximab or 1 μM of ERK inhibitor (Sigma-Aldrich). Conditions were repeated in triplicate. At indicated time points, viable cells were quantified using the Cell Counter Kit 8 (CCK-8, Enzo Life Sciences) according to the manufacturer’s instructions. Absorbance was measured at 450 nm using SpectraMax i3.

### Statistical Analysis

Tumor response curves were generated by plotting mean tumor volume and standard deviation. Log-transformed tumor growth over time were modeled and compared between treatment groups using linear mixed-effects models and Tukey method was used to adjust for p values in *post hoc* pairwise comparison. Surviving fraction was analyzed using a linear mixed model with logarithm base 10 transformation of survival colonies, in which individual samples were modeled as a random effect, while treatment group and radiation dose and their interaction were modeled as fixed effects. The *post hoc* pairwise comparison analysis was conducted with Tukey adjustment for p-values of the two-way interaction effect between radiation dose and treatment. Observed differences among groups from IHC, qPCR and flow cytometry were analyzed using ANOVA and Tukey’s method for multiple comparison was used to adjust for p values in *post hoc* pairwise comparison. Student’s t test was used for two-sample comparison. Mouse survival curves were generated using the Kaplan-Meier method. BH’s method for p values adjustments was used to assess the multiple comparisons of survival curves. All statistical tests were two-sided, and 5% (p < 0.05) was set as the level of significance. Statistical analysis was done in R 3.4.2. All experiments were replicated to confirm reported observations and data from the first of replicate studies is shown.

## Results

### Murine HNSCC Cells That Express huEGFR at the Plasma Membrane Are Resistant to Cetuximab Effects on Cell Viability and EGFR Signaling

To enable testing of the potential immune-based effects of cetuximab (anti-huEGFR antibody) against HNSCC tumor cells, we generated syngeneic murine models of HNSCC that express huEGFR. Because cetuximab does not recognize or antagonize murine EGFR, we expected that these models would be resistant to the effects from cetuximab that are dependent on blockade of EGFR signaling. We hypothesized, therefore, that syngeneic murine HNSCC tumor models expressing huEGFR would enable us to evaluate immune-mediated effects of cetuximab, such as ADCC, without the potentially confounding effects of cetuximab on tumor cell viability and radiation sensitivity.

**Figure 1:**
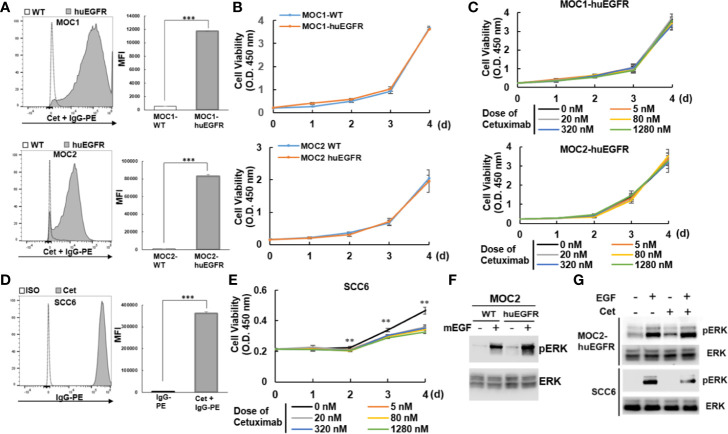
Murine MOC1 and MOC2 HNSCC cell lines expressing huEGFR at the plasma membrane are resistant to cetuximab effects on cell viability and EGFR signaling. **(A)** Cell surface expression of huEGFR in the MOC1 and MOC2 murine HNSCC cell lines (MOC1-, MOC2-huEGFR) was detected by flow cytometry using cetuximab (anti-huEGFR mAb). **(B)** Expression of huEGFR did not altered cell viability of MOC1 and MOC2 compared to parental cells (WT). **(C)** Cetuximab did not affect the viability of huEGFR-expressing MOC1 and MOC2. **(D)** The human HNSCC cell line, SCC6, over-expresses huEGFR at a level comparable to that of our murine models, as detected by flow cytometry using cetuximab as a primary antibody. **(E)** In contrast with our huEGFR-expressing murine HNSCC cells, treatment with cetuximab reduced the viability of human SCC6 cells. **(F)** Expression of huEGFR modestly increased mEGF-induced ERK phosphorylation in MOC2 cells. **(G)** Cetuximab did not affect mEGF-mediated ERK phosphorylation in MOC2-huEGFR, whereas cetuximab inhibited ERK phosphorylation in SCC6. (*p < 0.05, **p < 0.01, ***p < 0.001, at least two independent experiments).

To begin, we generated huEGFR-expressing MOC1 and MOC2 cell lines by viral transduction. MOC1 and MOC2 have been described previously, with MOC2 being more immunologically “cold” compared to MOC1 and characterized by low MHC1 expression and low levels of tumor infiltrating lymphocytes with a predominance of suppressive regulatory T cells (Tregs) (38, 39). Following transduction to express huEGFR, we confirmed that cetuximab was capable of binding MOC1- and MOC2-huEGFR cells, whereas cetuximab did not bind to WT MOC1 and MOC2 cells (**Figure 1A**). We observed that expression of huEGFR did not affected the viability of MOC1- and MOC2-huEGFR cells compared to WT MOC1 and MOC2 (**Figure 1B**). Given that cetuximab can inhibit the viability of huEGFR-expressing HNSCC cells (10), we tested whether cetuximab antagonized the viability of MOC1- or MOC2-huEGFR. We observed that the viability of MOC1- and MOC2-huEGFR tumor cells was not affected by cetuximab (**Figure 1C**). In contrast, using these same approaches we confirmed that cetuximab binds to and antagonizes the viability of human SCC6 HNSCC cells, which endogenously overexpress huEGFR (**Figures 1D, E**).

Next, we tested the effect of cetuximab on the activation of ERK, a downstream target of EGFR signaling (40, 41). We observed that expression of huEGFR in MOC2 cells resulted in a modest increase in murine EGF ligand-induced ERK phosphorylation compared to WT MOC2 (**Figure 1F**). While cetuximab suppressed EGF-stimulated ERK phosphorylation in human SCC6 cells as expected, it did not inhibit EGF-induced ERK phosphorylation on MOC2-huEGFR (**Figure 1G**), consistent with persistent mEGFR signaling in these cells in the presence of cetuximab. In similarly designed studies, we confirmed that mEGF increased ERK phosphorylation in MOC1-huEGFR cells compared to WT MOC1 (**Supplementary Figure 1A**). We further confirmed that despite no effect of cetuximab on the viability of MOC1- and MOC2-huEGFR, these cells remain sensitive to targeted inhibition of the EGFR signaling pathway when using a small molecule ERK inhibitor (**Supplementary Figure 1B**). These results suggest that because of endogenous expression of mEGFR, cetuximab binding to huEGFR does not affect EGF-induced mitogenic signaling in MOC1- and MOC2-huEGFR cells.

### huEGFR-Expressing Murine HNSCC Cells Are Not Sensitized to Radiation by Cetuximab but Upregulate Type I Interferon and NKG2D Ligands Following Radiation

**Figure 2:**
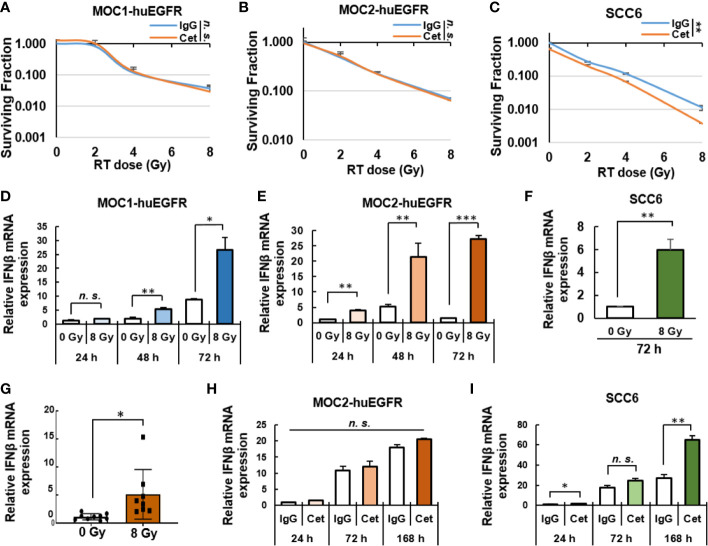
Cetuximab does not affect the radiosensitivity of MOC1- and MOC2-huEGFR cells but radiation induces a type I interferon response in these cells. **(A–C)** Cetuximab did not affect the radiosensitivity on huEGFR-expressing MOC1 and MOC2 but does in SCC6, as measured by *in vitro* clonogenic assays performed in the presence of human-IgG control or cetuximab (0.5 μg/ml). **(D–F)** 8 Gy radiation induced *Ifnβ* expression in MOC1- and MOC2-huEGFR cells and in SCC6 cells *in vitro* as determined *via* qPCR at 24, 48, and 72 h after radiation. **(G)** In MOC2-huEGFR tumors, local radiation (8 Gy) increased bulk tumor mRNA expression of *Ifnβ* compared to 0 Gy sham radiation. **(H)** In MOC2-huEGFR cells treated with 8 Gy radiation *in vitro*, cetuximab did not impact the magnitude or time course of radiation-induced *Ifnβ* expression. **(I)** In SCC6 cells, however, cetuximab did increase the effect of radiation in inducing *Ifnβ* expression at 24 and 168 h after radiation. (mean ± SEM, *p < 0.05, **p < 0.01, ***p < 0.001, *n. s.*, not significant, Student T-test, at least two independent experiments).

We evaluated the potential impact of cetuximab on the intrinsic radiosensitivity of MOC1- and MOC2-huEGFR cells. We did not detect any effect of cetuximab on the sensitivity of MOC1- or MOC2-huEGFR cells (**Figures 2A, B**) or on WT MOC1 and MOC2 cells (**Supplementary Figure 2**). Consistent with prior reports (10), cetuximab increased the radiosensitivity of human SCC6 cells (**Figure 2C**). These observations support the critical role of EGFR signaling blockade in the known effect of cetuximab on DNA damage response and on tumor cell sensitivity to radiation (10). Consistent with this, we observed that cetuximab does not affect the production of γH2AX, a marker of DNA double-strand breaks (15), following radiation of MOC2-huEGFR cells (**Supplementary Figure 1C**).

Activation of a type I interferon response in tumor cells following radiation is critical to the role of radiation in enhancing response to immunotherapies including anti–PD-1/PD-L1 checkpoint blockade (42). To evaluate radiation-induced effects on the immunogenicity of HNSCC cells *in vitro*, we used qPCR to measure changes in the expression of *Ifnβ* in murine MOC1- and MOC2-huEGFR and human SCC6 cells exposed to 8 Gy of radiation. We observed that radiation significantly increased *Ifnβ* expression in each of these cell lines compared to non-radiated controls (**Figures 2D–F**). We similarly evaluated the effect of radiation on the expression of *Ifnβ* in MOC2-huEGFR tumors *in vivo*. For this, mice bearing MOC2-huEGFR tumors (200 mm^3^) were treated with 8 Gy or sham radiation. After 5 days, the tumors were resected, mRNA was isolated, and gene expression was quantified by qPCR. Consistent with prior reports on the effects of radiation therapy in other tumor models (42, 43), we detected increased *Ifnβ* expression in MOC2-huEGFR tumors treated with 8 Gy as compared to the non-radiated controls (**Figure 2G**). We observed no effect of cetuximab on the induction of *Ifnβ* expression in MOC2-huEGFR cells following 8 Gy radiation delivered *in vitro* (**Figure 2H**). In contrast, cetuximab increased the induction of *Ifnβ* expression in human SCC6 cells following 8 Gy radiation *in vitro* (**Figure 2I**), suggesting that the radiosensitizing effects of cetuximab may further enhance the type I interferon response induced by radiation in tumor cells that are sensitive to cetuximab-mediated blockade of EGFR signaling.

### Cetuximab and Radiation Cooperate to Enhance the ADCC Anti-Tumor Immune Response

**Figure 3:**
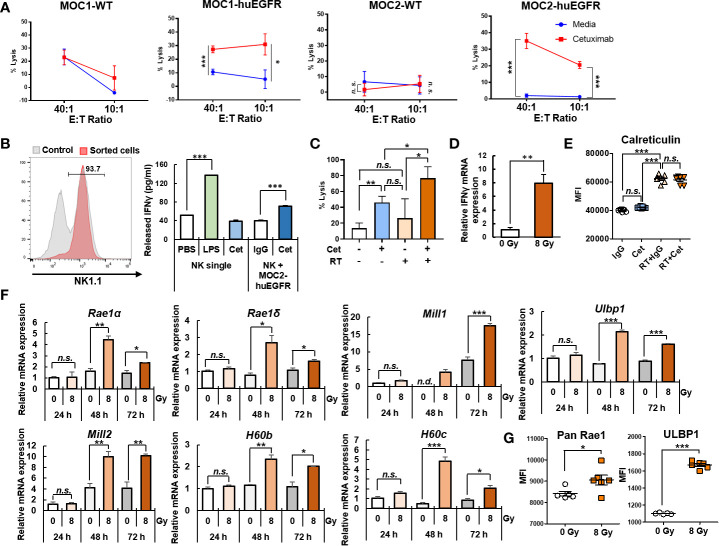
Radiation enhances cetuximab-mediated ADCC and activation of NK cells. **(A)** Cetuximab (0.5 μg/ml) induced ADCC against huEGFR-expressing MOC1 and MOC2 cells but not against WT MOC1 and MOC2 cells in a ^51^Cr-release assay. Percent of lysis among target tumor cells is presented. **(B)** Cetuximab increased IFNγ release in media collected after 24 h co-culture of murine NK cells (sorted from splenocytes by MACS) and MOC2-huEGFR in the presence of cetuximab, but not in the presence of non-specific human IgG, as measured by ELISA. Cetuximab alone did not affect IFNγ production compared to PBS (negative control), whereas LPS (positive control) did. **(C)** Cetuximab-mediated ADCC response was significantly increased against MOC2-huEGFR cells treated with 8 Gy radiation, compared to non-radiated MOC2-huEGFR cells, as determined by ^51^Cr-release assay on day 3 after radiation or sham radiation. In contrast, radiation did not significantly alter cytotoxicity when combined with non-specific IgG control. **(D)** mRNA expression of *Ifnγ* was increased in NK cells sorted from murine spleen and co-cultured for 12 h with radiated (8 Gy) MOC2-huEGFR cells in the presence of cetuximab (0.5 μg/ml), as compared to non-radiated (0 Gy) MOC2-huEGFR cells in the presence of cetuximab, as determined by qPCR. **(E)** As measured by flow cytometry, radiation increased calreticulin expression at the plasma membrane of MOC2-huEGFR 72 h after radiation, but cetuximab (0.5 μg/ml) did not alter the level of calreticulin at the plasma membrane in these cells, either in with or without radiation (8 Gy). **(F, G)** 8 Gy radiation induced increased mRNA expression of multiple NKG2D ligands by qPCR (after 48 h to 72 h) and protein expression of the NKG2D ligands RAE1 and ULBP1 at the plasma membrane by flow cytometry (after 72 h of culture) in MOC2-huEGFR tumor cells. (mean ± SEM, *p < 0.05, **p < 0.01, ***p < 0.001, *n. s.*, not significant, *n. d*., not detectable, Student T-test, at least two independent experiments).

We hypothesized that despite the lack of cetuximab effect on viability or radiosensitivity of MOC1- and MOC2-huEGFR cells, the expression of huEGFR at the plasma membrane of these cells (**Figure 1A**) could render them susceptible to cetuximab-mediated ADCC. Furthermore, given the potential for type I IFN to enhance the activity of ADCC effector cells (44–46), we hypothesized that radiation might augment cetuximab-mediated ADCC. Importantly the MOC1- and MOC2-huEGFR HNSCC models allow us to test for such a cooperative interaction in the absence of confounding effects of cetuximab on tumor cell viability and radiosensitivity. WT or huEGFR-expressing MOC1 and MOC2 cells were co-cultured with PBMCs and examined using a ^51^Cr-release assay to evaluate for tumor-specific ADCC elicited by cetuximab (**Figure 3A**). Cetuximab induced ADCC against huEGFR-expressing MOC1 and MOC2 cells, and this effect was not seen with WT MOC1 or MOC2 cells. These effects correlated with an increase in IFNγ production in sorted NK cells when co-cultured with MOC2-huEGFR and cetuximab or with positive control lipopolysaccharide (LPS), but not when NK cells were co-cultured with cetuximab alone or with MOC2-huEGFR cells alone (**Figure 3B**).

Importantly, we observed that irradiation of MOC2-huEGFR cells enhanced the capacity of cetuximab to elicit ADCC against these targets compared to non-radiated MOC2-huEGFR cells (**Figure 3C**). In agreement with this, we observed increased NK cell expression of the activation marker *Ifnγ* following co-culture of sorted NK cells with cetuximab and radiated MOC2-huEGFR cells, compared to co-culture with cetuximab and non-radiated MOC2-huEGFR cells (**Figure 3D**).

We evaluated potential mechanisms whereby radiation might contribute to an enhanced ADCC response. Radiation is known to induce immunogenic tumor cell death (47, 48). We evaluated the plasma membrane translocation of calreticulin as a marker of radiation-induced immunogenic cell death (49) in MOC2-huEGFR cells treated with radiation and/or cetuximab using flow cytometry (**Figure 3E**). Consistent with prior studies, we observed that radiation increased the expression of calreticulin at the cell surface in MOC2-huEGFR cells. However, in these cells, in which cetuximab does not affect viability or radiosensitivity, we did not observe an effect of cetuximab on this marker of immunogenic cell death either alone or with radiation. Many tumors express NKG2D ligands and these are upregulated by cell stress and enhance the susceptibility of cells to elimination by cytotoxic NK cells (50, 51). We therefore tested whether the enhanced ADCC response observed against MOC2-huEGFR cells following radiation might be associated with increased expression of NKG2D ligands. Following 8 Gy radiation of MOC2-huEGFR, *Rae1α/δ*, *Mill1/2*, *H60b/c*, and *Ulbp1* all exhibited significantly increased gene transcription by qPCR (**Figure 3F**) and we confirmed increased expression of RAE1 and ULBP1 proteins at the plasma membrane in these cells by flow cytometry (**Figure 3G**). Transcription of other NKG2D ligands including *Rae1β*, *Rae1γ*, and *H60a* was not detected in MOC2-huEGFR tumors. These data indicate that radiation promotes cetuximab-mediated ADCC and this may result in part from a novel effect of radiation enhancing the susceptibility of tumor cells to NK cell-mediated cytotoxicity by increasing expression of NKG2D ligands.

### NK Cell-Dependent Increase in the Local Anti-Tumor Effect of Radiation Therapy by Cetuximab

**Figure 4:**
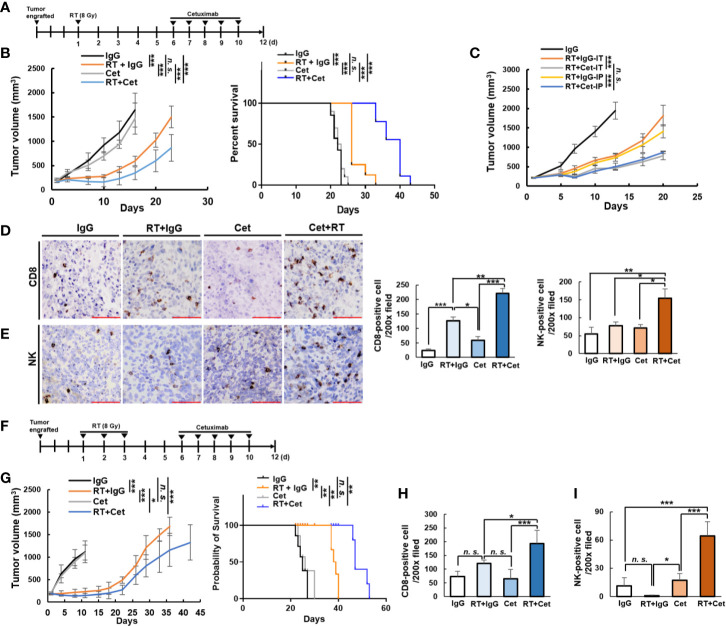
Cooperative therapeutic interaction between cetuximab and radiation in the MOC2-huEGFR model. **(A)** Mice bearing MOC2-huEGFR tumors were treated with radiation (8 Gy) or sham radiation (0 Gy), and non-specific human IgG or cetuximab (50 μg) were injected IT on days 6–10 after radiation. **(B)** Cetuximab alone showed no effect on tumor growth compared to non-specific control IgG and radiation slightly delayed tumor growth. When given together, radiation + cetuximab significantly increased this delay in tumor growth and increased overall survival (*n* = 7–10/group). **(C)** No difference was observed in tumor response using either systemic delivery of cetuximab by IP injection or local delivery by IT injection when MOC2-huEGFR tumor-bearing mice were treated with 8 Gy tumor radiation or sham radiation on day 1 and non-specific human IgG or cetuximab (50 μg) on days 6–10 (*n* = 4/group). **(D, E)**, Tumor infiltrating CD8+ T cells and NK cells were detected by immunohistochemistry in tumors harvested on day 12 after 8 Gy radiation or sham radiation. Scale bar indicates 100 µm. **(F)** Mice bearing MOC2-huEGFR tumors were treated with 8 Gy radiation daily from days 1 to 3. Non-specific human IgG or cetuximab (50 μg) were injected IT on days 6–10 after radiation. **(G)** Radiation (8 Gy × 3 fractions) + cetuximab significantly increased tumor growth delay and increased overall survival (*n* = 5/group). **(H, I)** Tumor infiltrating CD8+ T cells and NK cells were increased following combined radiation (8 Gy × 3 fractions) + cetuximab, however the degree of NK infiltrate in tumors appeared to be reduced at this time point compared with 8 Gy × 1 fraction in E. (mean ± STDEV, *p < 0.05, **p < 0.01, ***p < 0.001, *n. s.*, not significant, multiple comparison by ANOVA with *post hoc* Tukey, at least three independent animal experiments).

To test for cooperative immune-mediated anti-tumor effects of radiation and cetuximab, we implanted MOC2-huEGFR tumors in C57BL/6 mice. When the average tumor volume reached 150-200 mm^3^, tumors were treated with local radiation (8 Gy) or sham radiation and daily IT injections of cetuximab or non-specific control human IgG antibody (50 μg/injection) on days 6–10 after radiation (**Figure 4A**). Cetuximab alone showed no significant effect on tumor growth compared to non-specific control human IgG and local radiation alone resulted in mild tumor growth delay (**Figure 4B**). Compared to these treatments, the combination of radiation and cetuximab resulted in significantly increased tumor growth delay and improved overall survival (**Figure 4B**). We evaluated the potential impact of different routes of cetuximab delivery on this cooperative therapeutic interaction with radiation. Both intraperitoneal (35) and IT injections of cetuximab delayed the tumor growth and were not significantly different from one another (**Figure 4C**).

To assess differences in the tumor immune infiltrate among mice receiving radiation and/or cetuximab, tumor tissue was collected on day 12 after radiation from a separate cohort of mice and immunohistochemistry was performed. Consistent with prior studies, we observed a modest increase in CD8+ T cells in tumors treated with radiation alone (19), and this effect was enhanced in tumors treated with the combination of radiation and cetuximab (**Figure 4D**). In contrast, radiation and cetuximab treatments alone did not affect tumor infiltration by NK cells, but the combination of cetuximab and radiation significantly increased tumor infiltration by NK cells (**Figure 4E**). These results demonstrate that pairing cetuximab with radiation increases MOC2-huEGFR tumor response, despite no effect of cetuximab on the viability or radiosensitivity of this tumor model (**Figures 1B** and **2B**) and this augmented response is associated with increased tumor infiltration by both CD8+ T cells and NK cells in MOC2-huEGFR tumors.

Previous studies indicate that in some settings three fractions of 8 Gy radiation may be more effective in activating a type I IFN response and anti-tumor immune response compared to a single 8 Gy fraction (42). We evaluated the impact of cetuximab when combined with an 8 Gy × 3 fraction radiation regimen (**Figure 4F**). We observed that this combination treatment resulted in enhanced tumor regrowth delay and a significant increase in overall survival compared to cetuximab alone or 8 Gy × 3 fractions of radiation alone (**Figure 4G**) as well as significantly increased tumor infiltration by CD8+ T cell and NK cells (**Figures 4H, I**). However, the number of tumor-infiltrating NK cells was lower in cohorts treated with 8 Gy × 3 compared to 8 Gy × 1 (**Figures 4E, I**). Interestingly, we observed that upregulation of NKG2D ligands in MOC2-huEGFR tumors following 8 Gy × 3 fractions of radiation was comparable to or greater than that achieved by a single 8 Gy fraction (**Supplementary Figures 3A, B**). However, expression of MHCI, which is inhibitory to NK cells, was increased to a greater extent following 8 Gy × 3 fractions as compared to a single 8 Gy fraction (**Supplementary Figures 3C, D**).

### NK Cells, Host FcγR, and huEGFR Are Required for the Cooperative Interaction of Radiation and Cetuximab Therapy

**Figure 5:**
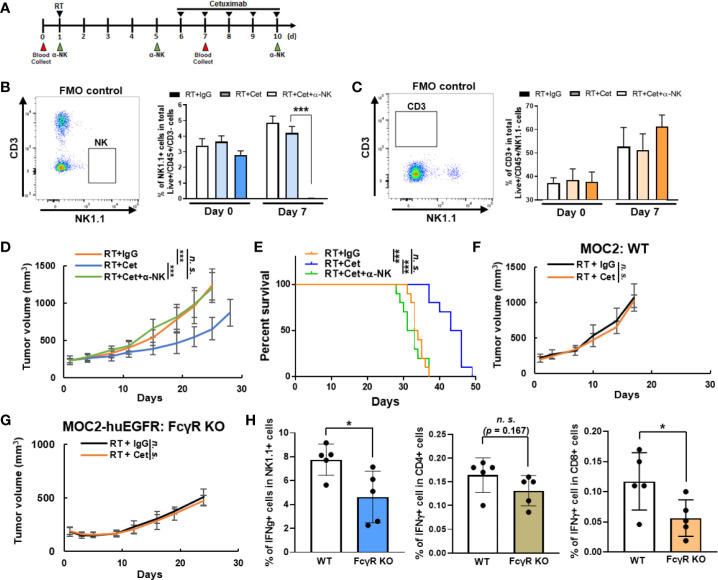
The cooperative therapeutic interaction between radiation and cetuximab in MOC2-huEGFR tumors requires huEGFR, host expression of FcγR, and NK cells. **(A)** Intratumoral cetuximab treatments, with or without intraperitoneal anti–NK1.1 antibody (50 μg), were administrated on indicated day after tumor radiation. **(B, C)**, Peripheral blood was collected to confirm the selective depletion of NK cells (mean ± STDEV, *p < 0.05, **p < 0.01, ***p < 0.001, Student T-test). **(D, E)** The combination of cetuximab and radiation did not improve anti-tumor response or overall survival in mice depleted of NK cells (*n* = 10/group). **(F, G)**, Treatment was administered as per **Figure 4A** (*n* = 4–5/group). **(F)** Expression of huEGFR was required to elicit anti-tumor response to combined radiation (8 Gy) and cetuximab treatment. **(G)** Combined radiation (8 Gy) and cetuximab did not improve response compared to radiation alone in MOC2-huEGFR tumors when delivered in host syngeneic mice that were lacking FcγR expression. **(H)** Co-culture of splenocytes from WT or FcγR KO mice with radiated MOC2-huEGFR (8 Gy) in the presence of cetuximab (2 µg/ml) for 24 h results in increased expression of IFNγ in NK and CD8+ T cells among WT but not FcγR-deficient splenocytes. Expression of IFNγ was analyzed using flow cytometry (mean ± STDEV, *p < 0.05, ***p < 0.001, *n. s.*, not significant, Student T-test, at least three independent animal experiments).

We hypothesized that the effect of cetuximab in augmenting anti-tumor response to local radiation in MOC2-huEGFR tumors was mediated, at least in part, by NK cells. To test this, we treated mice bearing MOC2-huEGFR tumors with 8Gy and daily IT injections of cetuximab as in **Figure 4A** and compared the effect of this treatment with that observed in a cohort of mice depleted of NK cells (**Figure 5A**). We confirmed that IP administration of anti-NK1.1 antibody depleted NK cells but not CD3+ cells (**Figures 5B, C**). Depletion of NK cells resulted in a complete loss of the cooperative therapeutic interaction between cetuximab and radiation in treating MOC2-huEGFR tumors (**Figures 5D, E**). Similarly, we tested the necessity of tumor cell expression of huEGFR in this cooperative therapeutic interaction by treating mice bearing WT MOC2. We observed no differences between treatment with radiation and cetuximab versus radiation and non-specific control human IgG in these tumors, indicating that huEGFR expression was necessary for the cooperative therapeutic interaction between cetuximab and radiation *in vivo* (**Figure 5F**). Using FcγR-deficient C57BL/6 mice, we also confirmed that the cooperative therapeutic effects of radiation and cetuximab require expression of FcγR on the host-animal’s immune cells (**Figure 5G**). Notably, co-culture of splenocytes with radiated MOC2-huEGFR cells in the presence of cetuximab resulted in increased expression of IFNγ in not only NK cells but also in CD8+ T cells (**Figure 5H**). This activation of both NK and T cells was dependent upon splenocyte expression of FcγR (**Figure 5H**). This indicates that although radiation induced NKG2D ligand expression in MOC2-huEGFR, direct effector engagement of these cells *via* antibody-FcγR binding is required to activate NK cells. This further suggests that *in vitro* activation of innate FcγR-expressing cells could secondarily activate adaptive effector T cells, which do not express FcγR. Collectively, these results demonstrate an NK-cell mediated, FcγR-dependent, cooperative therapeutic interaction between to local radiation and cetuximab in huEGFR-expressing tumors.

### Radiation Combined With Cetuximab Augments Response to Anti–PD-L1 Checkpoint Inhibition

**Figure 6:**
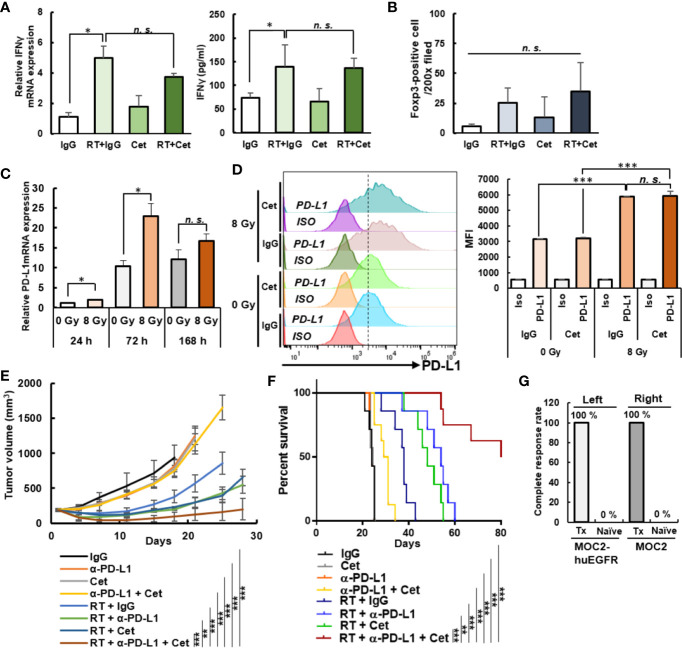
Anti-tumor immune response to anti–PD-L1 immune checkpoint blockade is enhanced by combined treatment with radiation and cetuximab in MOC2-huEGFR tumors. **(A)** Combined radiation (8 Gy) and cetuximab therapy did not increase *Ifnγ* expression in bulk tumor compared to radiation alone on day 12 after radiation as determined by qPCR (left) and ELISA (right)—despite greater infiltration of these tumors by NK and T cells (see **Figure 4D**) (mean ± STDEV, *p < 0.05, **p < 0.01, ***p < 0.001, multiple comparison by ANOVA with *post hoc* Tukey). **(B)** FOXP3+ cells were analyzed from tumor immunohistochemistry of **Figure 4D**. A non-significant trend was observed toward an increase in FOXP3+ cells among tumors treated with radiation or radiation plus cetuximab (mean ± STDEV, *p < 0.05, **p < 0.01, ***p < 0.001, multiple comparison by ANOVA with *post hoc* Tukey). **(C)** Radiation induced *Pd-l1* expression in MOC2-huEGFR cells treated with 8 Gy, as measured by qPCR (mean ± SEM, *p < 0.05, **p < 0.01, ***p < 0.001, Student T-test). **(D)** MOC2-huEGFRs were treated with 8 Gy radiation and stimulated with non-specific human IgG or cetuximab (2 μg/ml) 3 days before flow cytometry analysis. PD-L1 expression was observed to increase following radiation with no effect noted from cetuximab on this response (mean ± STDEV, *p < 0.05, **p < 0.01, ***p < 0.001, multiple comparison by ANOVA with *post hoc* Tukey). **(E)** Anti–PD-L1 antibody increased anti-tumor response elicited by radiation and cetuximab in MOC2-huEGFR tumor-bearing mice resulting in tumor regression and **(F)** a durable survival benefit with this triple combination compared to single or dual agent control treatments (*n* = 7–10/group). **(G)** Naïve (*n* = 3) and disease-free mice (Tx, *n* = 3) were rechallenged by subcutaneous right flank MOC2 cell injection and left flank MOC2-huEGFR cell injection. The percentage of complete response is shown (*p < 0.05, **p < 0.01, ***p < 0.001, *n. s.*, not significant, at least three independent animal experiments).

In MOC2-huEGFR tumors treated with combined cetuximab and radiation, we evaluated markers of immune activation and suppression. Even though combination therapy promoted CD8+ T cell and NK cell infiltration compared to radiation alone (**Figure 4D**), we observed no differences in Ifnγ gene expression or IFNγ cytokine production in tumors treated with radiation alone or radiation plus cetuximab (**Figure 6A**). We hypothesized that suppressive features in the immunologically “cold” MOC2-huEGFR tumor microenvironment or on these tumor cells might be blunting the activation of adaptive anti-tumor immunity among the increased number of tumor-infiltrating lymphocytes observed after combined radiation and cetuximab.

By immunohistochemistry we quantified FOXP3+ cells, which include regulatory T cells (Tregs), but we identified only a non-significant trend toward an increase in this population at day 12 following radiation or radiation plus cetuximab (**Figure 6B**). Following *in vitro* radiation of MOC2-huEGFR cells, however, we did identify a significant increase in the mRNA expression of programed death-ligand 1 (Pd-l1) (**Figure 6C**). This resulted in a radiation-induced increase in the cell surface expression of PD-L1 and this was not altered when radiation was delivered in the presence of cetuximab (**Figure 6D**). We hypothesized that this radiation-induced expression of PD-L1 on tumor cells might blunt the activation of an adaptive anti-tumor immune response *in vivo* following combined treatment with radiation and cetuximab. To test this, we administered systemic anti–PD-L1 therapy (200 µg IP, days 0, 4 and 7 after radiation) in combination with radiation and cetuximab in syngeneic mice bearing MOC2-huEGFR tumors. We observed enhanced tumor regression and increased overall survival in mice treated with the combination of radiation, cetuximab, and anti–PD-L1 antibody as compared to mono- or dual combinations of these treatments (**Figures 6E, F**). This combined treatment led to complete tumor regression in 30% (n = 3/10) of mice bearing the immunologically cold, MOC2-huEGFR tumor, whereas no complete response was observed in tumor-bearing naive mouse (**Figure 6G**).

Among these mice rendered disease-free, we tested for an adaptive anti-tumor memory response by re-engrafting these mice and age-matched naïve controls with MOC2 and MOC2-huEGFR in the upper right flank and upper left flank (both outside of the prior treatment field), respectively. We observed that all disease-free mice rejected both the MOC2 and MOC2-huEGFR cells compared with 100% engraftment among control mice. These data suggest a potent adaptive anti-tumor memory response against antigen(s) shared by MOC2-huEGFR and MOC2, consistent with an *in situ* vaccination effect.

## Discussion

We generated huEGFR-expressing syngeneic murine models of HNSCC for the purpose of evaluating immune-mediated therapeutic interactions between radiation and the anti-huEGFR antibody, cetuximab. These murine models uniquely enable evaluation of such immune-mediated mechanisms because they are not sensitive to the potentially confounding effects of cetuximab on tumor cell viability or radiosensitivity. This results from the inability of cetuximab to bind and antagonize mEGFR. In their persistent expression of huEGFR at the plasma membrane but lack of sensitivity to anti-proliferative and radiosensitizing effects of cetuximab, these huEGFR-expressing murine tumor models are phenotypically analogous to human HNSCC tumor cells with acquired cetuximab resistance (52, 53). We acknowledge many differences between our murine models and clinically acquired resistance to cetuximab in human HNSCC. Notably, acquired resistance to cetuximab often results from activation of alternative ErbB family signaling pathways leading to persistent ERK activation (35). In contrast, our murine models achieve this through persistent ERK activation downstream of mEGFR. However, with an understanding of such limitations, these huEGFR-expressing syngeneic murine tumor models can serve as a unique tool for evaluating immune-mediated mechanisms of cetuximab and the interaction of these mechanisms with radiation or other therapeutic modalities in syngeneic mice. Given the known and potentially confounding effects of cetuximab in inhibiting EGFR+ HNSCC tumor cell viability and in sensitizing these cells to radiation (7, 8, 10), we are not aware of any alternative syngeneic HNSCC model that would allow for testing of the interaction between radiation and cetuximab-mediated ADCC.

In patients with HNSCC, an adaptive immune cell tumor infiltrate is associated with improved treatment outcomes (54–56). Here, we observe that cetuximab alone does not alter NK cell infiltration of the MOC2-huEGFR tumor or reduce growth of this tumor, but is capable of eliciting ADCC against HNSCC tumor cells independent of its roles in blocking EGFR signaling or enhancing radiosensitivity. When combined with local radiation, cetuximab increased both NK cell and CD8+ T cell tumor infiltration *in vivo* and enhanced ADCC response to cetuximab *in vitro*. This may result from effects of radiation that enhance the susceptibility of tumor cells to ADCC, including activation of a type I IFN response, induction of immunogenic cell death in neighboring tumor cells, and increased expression of NKG2D ligands. We observed that radiation gradually increased IFNβ in MOC2-huEGFR out to 168 h and this effect was not modified by the presence of cetuximab. Therefore, *in vivo*, we hypothesized that the susceptibility of radiated tumor cells to ADCC would be highest at a delayed time point (when the type I IFN response was maximal). This expectation was also influence by our prior observations testing the timing of radiation and tumor specific antibody response, where we observed greater anti-tumor immune effect when tumor-specific antibody delivery was delayed rather than concurrent with radiation (19). Indeed, when we combined radiation and delayed administration of cetuximab (days 6–10 after radiation) *in vivo* for treatment of our MOC2-huEGFR HNSCC model, we observed improved tumor response and overall survival.

We did not observe curative treatment effects from combined radiation and cetuximab in the spontaneously metastatic MOC2-huEGFR HNSCC model. This suggests that radiation and cetuximab did not fully stimulate activation of tumor-specific T cells, perhaps due to simultaneous activation of suppressive mechanisms. Indeed, we observed increased *Pd-l1* expression in MOC2-huEGFR cells following this treatment regimen *in vivo* (**Figure 6C**). Studies examining tumor surface PD-L1 expression have suggested that IFNβ and IFNγ produced from immune cells stimulate PD-L1 expression on tumors (57, 58). Another group observed that radiation elicits PD-L1 expression on melanoma and glioblastoma (59). In the present study, we observed that radiation increases IFNβ and PD-L1expression in murine models of HNSCC. We hypothesize that in these tumor models IFNβ production, induced in tumor cells by radiation, increases PD-L1 expression through autocrine and/or paracrine signaling mechanisms. Consequently, increased PD-L1 in these tumors may blunt to development or effect of an adaptive immune response following radiation and cetuximab. This may explain the benefit of anti–PD-L1 therapy when added to this radiation and cetuximab combination treatment, despite no apparent therapeutic efficacy of anti–PD-L1 when used alone in this immunologically “cold” tumor model.

PD-1/PD-L1 engagement is a well-known immune checkpoint for T cells and recent studies also show inhibitory effects of PD-1/PD-L1 on NK cell activation and viability (60). Inhibition of the PD-1/PD-L1 axis in HNSCC has resulted in a ~20% response rate and improved overall survival and anti–PD-1 therapy is now approved for frontline treatment of recurrent or metastatic HNSCC (17). In the immunologically “cold” MOC2-huEGFR model (39), we found that anti–PD-L1 monotherapy does not elicit an anti-tumor response (**Figure 4C**), despite gradually increased endogenous *Pd-l1* expression on growing tumor. However, by enhancing tumor cell susceptibility to NK cell killing and by increasing tumor infiltration and activation of NK cells, the combination of radiation and cetuximab therapy triggers recruitment and activation of CD8+ T cells, priming an adaptive response to “cold” tumors and enabling durable tumor eradication when combined with anti–PD-L1 therapy. With this combination treatment, 100% of mice exhibited anti-tumor response and 30% were cured. Unlike the combination of radiation and cetuximab alone, the adaptive immune response unleashed by combination with anti–PD-L1 conveyed immunologic memory to those mice that were cured and this adaptive response was equally effective against huEGFR+ or huEGFR-deficient variants of the eradicated tumor line. We speculate that we do not observe 100% cure among mice treated with radiation, cetuximab, and anti–PD-L1 checkpoint blockade due to the effects of additional mechanisms of immune inhibition, potentially including alternative immune checkpoint receptor-ligand interactions, and we will further evaluate approaches to overcoming these in future studies.

In support of the generalizability of our observations, we have previously reported preclinical studies demonstrating a therapeutic interaction between radiation and tumor-specific anti-GD2 antibody in murine models of melanoma and neuroblastoma (19). That effect was also NK-cell dependent. However, anti-GD2 antibody is not commonly delivered in conjunction with radiotherapy. On the other hand, cetuximab is the only tumor-specific antibody that is specifically approved for concurrent use with radiotherapy. This is based on a prior randomized clinical study that demonstrated improved overall survival in patients with locally advanced HNSCC treated with cetuximab and radiation, as compared to radiation alone (11). This effect has been thought to result predominantly from effects of cetuximab on tumor cell viability and radiosensitivity (10, 61–64). Our data now suggest that at least a component of this proven cooperative therapeutic effect may be immune-mediated.

In our prior study of the interaction of radiation and anti-GD2 antibodies (19), we did also evaluate the interaction of radiation and cetuximab, demonstrating a therapeutic effect against cetuximab-resistant human HNSCC tumor cells that expressed huEGFR at the cell surface. However, due to a lack of suitable syngeneic murine models at that time, those studies were performed in immunodeficient nude mice that have NK cells but lack T cell immunity. This precluded evaluation of the potential mechanisms of interaction between innate and adaptive immunity following combined treatment with radiation and cetuximab and did not allow for testing of the potential benefit of combining this approach with additional immunotherapies including PD-1/PD-L1 checkpoint blockade. We developed the MOC1- and MOC2-huEGFR tumor models specifically to overcome these limitations and to enable these preclinical investigations of therapeutic mechanisms whereby the combination of radiation and cetuximab might elicit a more robust *in situ* vaccine effect and prime adaptive response to immune checkpoint blockade.

Our results are consistent with previous studies showing that FcγR is required to activate NK cells (44). Although some studies have demonstrated that stimulation of NKG2D can trigger activation of NK cells even in the absence of FcγR (45), we observed that in FcγR-deficient NK cells the exposure to cetuximab and radiated tumor cells did not effectively activate IFNγ expression, despite up-regulation of NKG2D ligands on the radiated tumor cells. Our data suggest that the increased production of IFNγ in NK cells exposed to cetuximab and radiated tumor cells may contribute to activation of CD8+ T cells, as this effect that was dependent upon antibody and FcγR expression. This suggests that cetuximab and potentially other tumor-specific antibodies may augment the *in situ* vaccine effect of radiation therapy (23, 30). Given the availability of tumor-specific antibodies for a wide variety of tumor types, this portends tremendous translational potential for combining radiation and tumor-specific antibodies to achieve greater local and systemic tumor control.

**Figure 7:**
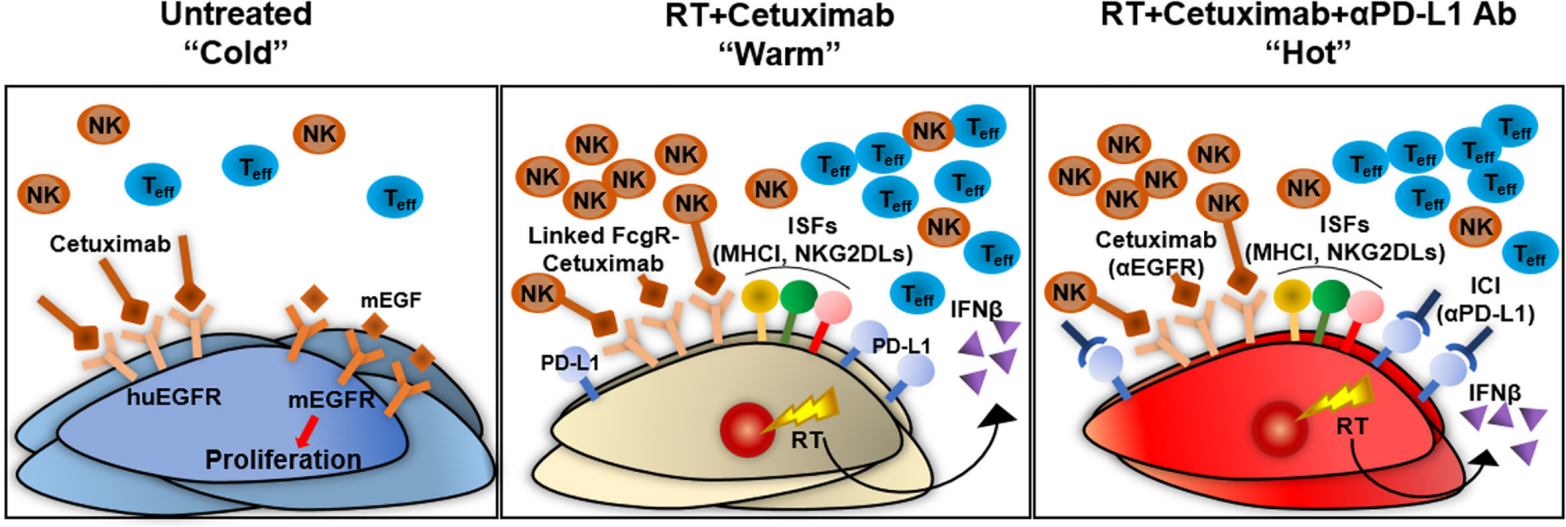
Summary of an *in situ* vaccine regimen combining radiation and cetuximab for the treatment of an immunologically “cold” HNSCC murine tumor model. Our huEGFR-expressing syngeneic murine HNSCC tumor models enable evaluation of immune-mediated mechanisms whereby cetuximab may elicit immune-dependent therapeutic effects because cetuximab is able to bind the huEGFR on these cells but does not antagonize mEGFR that is endogenously expressed. This eliminates experimentally confounding effects of cetuximab on EGFR signaling pathways. The MOC2-huEGFR tumor model is phenotypically analogous to an immunologically “cold” human HNSCC tumor with acquired cetuximab resistance. Cetuximab is capable of binding huEGFR on these cells but this does not affect cell viability or radiosensitivity. However, cetuximab is able to elicit ADCC against MOC2-huEGFR cells and this is enhanced when the tumor cells have been radiated. Radiotherapy alone can act as an *in situ* vaccination and induces tumor infiltration by CD8+ T cells and NK cells and increases surviving tumor cell susceptibility to both T and NK cell recognition and killing by increasing tumor cell expression of type I interferon and immune susceptibility markers including NKG2D ligands. This *in situ* vaccine effect of radiation is increased by combination with cetuximab, although this combination alone does not lead to durable tumor control. This results at least in part from increased expression of PD-L1 in the tumor following combined radiation and cetuximab treatment. Addition of anti–PD-L1 immune checkpoint inhibitor therapy to the combination of radiation and cetuximab overcomes this limitation and enables curative response with evidence of adaptive anti-tumor memory in some mice. These results indicate that the *in situ* vaccine effect of radiation may be augmented by combination with tumor-specific antibodies through more effective engagement of innate immune effectors that convert an immunologically cold tumor microenvironment to one that is immunologically “warm” and responsive to immune checkpoint blockade. ICI, immune checkpoint inhibitor; IFNβ, interferon beta; ISFs, immune susceptibility factors; mEGF, murine epidermal growth factor; MHCI, major histocompatibility complex I; NK, natural killer cells; RT, radiation; Teff, effector T cells.

Early phase clinical data has suggested safety for the combination of radiation, cetuximab, and anti–PD-1 checkpoint blockade (65), albeit with a fractionated approach to radiation therapy. Our results suggest that patients with metastatic HNSCC may benefit from treatment with combinations of radiation, cetuximab, and immune checkpoint blockade, including those patients with immunologically “cold” tumors not responding to anti–PD-1 therapy alone and those with acquired resistance to cetuximab but persistent tumor cell expression of huEGFR (**Figure 7**). This observation will lead an opportunity to optimize such treatment combinations, through future studies evaluating the varied dose-dependency of radiation effects on tumor cell expression of type I interferon, NKG2D ligands, PD-L1, and other markers of tumor cell susceptibility to innate and adaptive anti-tumor immunity.

## Data Availability Statement

The original contributions presented in the study are included in the article/**Supplementary Material**, further inquiries can be directed to the corresponding author.

## Ethics Statement

The animal study was reviewed and approved by the University of Wisconsin-Madison, Institutional Animal Care and Use Committee.

## Author Contributions

WJ was responsible for the primary conception and design of the experiments input from AE, CS, AJ, and ZM. Primary investigator ZM determined the experimental direction and provided study funding. CS, AJ, and JJ assisted with *in vitro* experiments for all studies. AE conducted gene transduction, ADCC, and tumor cell line quality check. BA, RS, and PC assisted with animal experiments including tumor measurements, survival rate, and IHC. Data analysis and interpretation were provided by AB, TL, YC, and KK. All authors contributed to the article and approved the submitted version.

## Funding

The authors would like to thank the NIH funding support from P30 CA014520, P50 DE026787, 1DP5OD024576, U01CA233102, T32GM008692 and TL1TR002375.

## Conflict of Interest

The authors declare that the research was conducted in the absence of any commercial or financial relationships that could be construed as a potential conflict of interest.
