# Supplementary material for: Tumor-Specific Antibody, Cetuximab, Enhances the In Situ Vaccine Effect of Radiation in Immunologically Cold Head and Neck Squamous Cell Carcinoma
Source: Front Immunol. 2020 Nov 12;11:591139. doi: 10.3389/fimmu.2020.591139 (PMC7689006; doi:10.3389/fimmu.2020.591139)
Supplement: Supplementary file 1 [file Table_1.docx]

Supplementary data

# Supplementary Figures 1.

**C.**

**A.**

**B.**


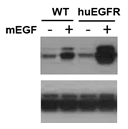


**pERK**

**ERK**

**MOC1**

**mEGF**

-

+

-

+

**WT**

**huEGFR**

***

***

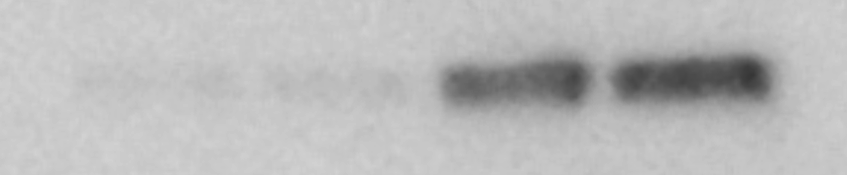


**Cet**

**RT**


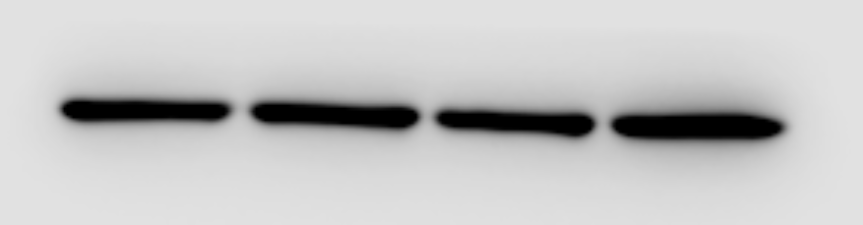


**GAPDH**

**γH2AX**

-

-

-

+

+

-

+

+

**MOC2-huEGFR**

**MOC1-huEGFR**

**Supplementary Figure 1.** *EGF-induced ERK phosphorylation is require for viability of MOC1- and MOC2-huEGFR cells.* (A) Expression of huEGFR in MOC1 cells promoted ERK phosphorylation by mEGF stimulation. (B) ERK inhibition resulted in decreased proliferation in MOC1-, MOC2-huEGFR cells. (C) In MOC2-huEGFR cells *in vitro* treatment with cetuximab did not affect RT-induced expression of γH2AX, a marker of radiation induced DNA damage.

# Supplementary Figures 2.


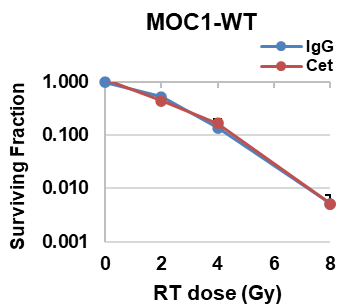


***n. s.***


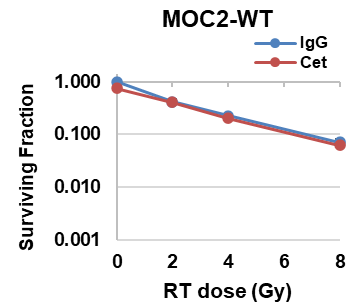


***n. s.***

**Supplementary Figure 2.** *Cetuximab does not affect the radio-sensitivity of WT MOC1 and MOC2 cells.* In vitro clonogenic assays demonstrate no effect of cetuximab on the radio-sensitivity of WT MOC1 and MOC2 cells.

Supplementary Figures 3.


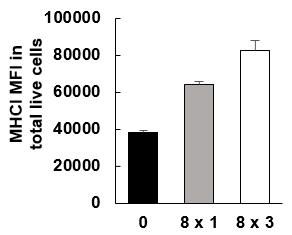

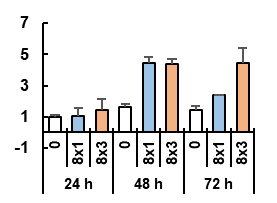

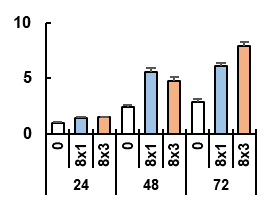


**Rae1α**


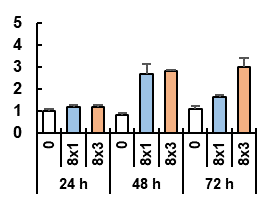


**Rae1δ**


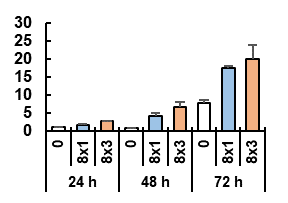


**Relative mRNA expression**

**Relative mRNA expression**

**Mill1**


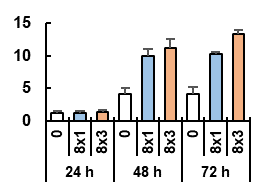


**Relative mRNA expression**

**Relative mRNA expression**


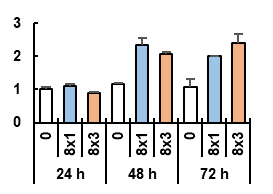


**Relative mRNA expression**

**Mill2**

**H60b**


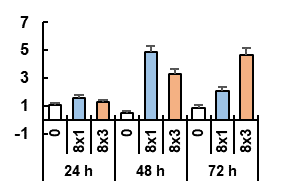


**Relative mRNA expression**

**H60c**


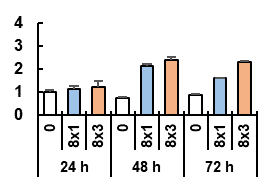


**Relative mRNA expression**

**ULBP1**

******

***n.s.***

***n.s.***

***n.s.***

***n.s.***

******

***n.s.***

******

***n.s.***

******

***n.s.***

*******

***n.s.***

******

***n.s.***

******

***n.s.***

***n.s.***

******

***n.s.***

******

***n.s.***

***n.s.***

*******

***n.s.***

*******

*******

***n.s.***

*******

***n.s.***

***n.s.***

******

******

***n.s.***

***n.s.***

*****

******

**(Gy)**


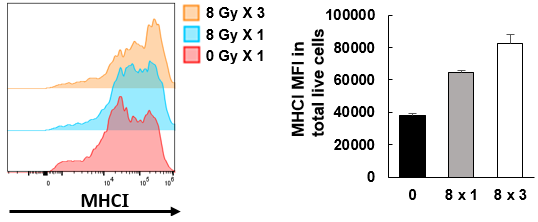


**(Gy)**

**D.**

**C.**

***n.s.***

***n. s.***

**72 h**

*******

*******

*******

******

*******

*******

*******

**Relative mRNA expression**

**MHCI**

**MHCI**

**8 Gy x 3**

**8 Gy x 1**

**0 Gy**

**(Gy)**

**(Gy)**


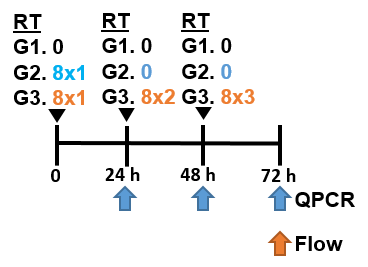


**A.**

**B.**

**Supplementary Figure 3.** *Effect of radiation dose on the expression of NKG2D ligands and MHCI.* (A) MOC2-huEGFR were treated with radiation (0 Gy, 8 Gy, or 8 Gy x3) on indicated days. (B) 8 Gy x 3 fractions resulted in comparable or greater induction of NKG2D ligand expression, compared to a single 8 Gy fraction, as determined by using qPCR. (C) 8 Gy x 3 stimulated a greater increase in MHCI mRNA expression compared to a single 8 Gy fraction, as determined by qPCR or (D) flow cytometry (mean±SEM, **p* < 0.05, ***p* < 0.01, ****p* < 0.001, multiple comparison by ANOVA with post-hoc Tukey).
